# Cell-in-cell structure in cancer: evading strategies from anti-cancer therapies

**DOI:** 10.3389/fonc.2023.1248097

**Published:** 2023-09-18

**Authors:** Kohei Okuyama, Hiromasa Fukushima, Tomofumi Naruse, Souichi Yanamoto

**Affiliations:** ^1^ Department of Periodontics and Oral Medicine, School of Dentistry, University of Michigan, Ann Arbor, MI, United States; ^2^ Rogel Cancer Center, University of Michigan, Ann Arbor, MI, United States; ^3^ Department of Oral and Maxillofacial Surgical Oncology, Graduate School of Medical and Dental Sciences, Tokyo Medical and Dental University, Tokyo, Japan; ^4^ Department of Clinical Oral Oncology, Nagasaki University Graduate School of Biomedical Sciences, Nagasaki University, Nagasaki, Japan; ^5^ Department of Oral Oncology, Graduate School of Biomedical and Health Sciences, Hiroshima University, Hiroshima, Japan

**Keywords:** cell-in-cell structure, entosis, phagocytosis, chemotherapy, targeted therapy, radiotherapy, immunotherapy

## Abstract

One of the regulated forms of cell death is the cell-in-cell (CIC) structure, in which a surviving cell is engulfed by another cell, a mechanism that causes the death of the engulfed cell by an adjacent cell. Several investigators have previously shown that the presence of CICs is an independent risk factor significantly associated with decreased survival in patients with various types of cancer. In this review, we summarize the role of CIC in the tumor microenvironment (TME), including changes and crosstalk of molecules and proteins in the surrounding CIC, and the role of these factors in contributing to therapeutic resistance acquisition. Moreover, CIC structure formation is influenced by the modulation of TME, which may lead to changes in cellular properties. Future use of CIC as a clinical diagnostic tool will require a better understanding of the effects of chemotherapy on CIC, biomarkers for each CIC formation process, and the development of automated CIC detection methods in tissue sections of tumor specimens.

## 1 Introduction

One of the regulated forms of cell death, entosis, called cell-in-cell (CIC) structures, in which viable cells are internalized into other cells, has been reported for over a century. It is a mechanism that targets cells for death following their engulfment by neighboring cells (1). CIC structures also can result from different types of nonautonomous cell death, such as entosis, cellular cannibalism, phagocytosis, encytosis, and emperipolesis. Entotic cells are autonomously killed by engulfing them through autophagic protein-dependent lysosomal digestion (2). Krajcovic et al. showed that entosis inhibited transformed growth by inducing cell death. However, this process promotes aneuploidy in host cells (3) and facilitates nutrient recovery by engulfing cells that promote tumor progression (4). Moreover, Wang et al. found that the presence of CIC is an independent risk factor significantly associated with decreased survival in patients with hepatocellular carcinoma, especially in patients with low-grade, early-stage cancer (5). Other studies have shown that the presence of CIC is associated with advanced-stage cancer and that this phenomenon is associated with genetic involvement (6).

The profile of CICs varies from tumor to tumor, which may indicate different malignant stages or inflammatory states. Phagocytosis is best known as the process by which one cell is taken up by another. Phagocytosis generally targets dead, dying, or pathogenic cells for engulfment and is driven by cytoskeletal rearrangements of the host cell in response to signals from the cell that are being taken up by the target cell. Target cells are then typically degraded by lysosomal enzymes within one hour (7). Sun et al. also described that entosis can act as a form of cellular competition, where the engulfment of loser cells by neighboring winners can promote clonal selection within heterogeneous tumor cell populations, and the competition is driven by a mechanical differences between softer and stiffer cells, where the stiffer cells are eliminated by the softer cells (8). In contrast to phagocytosis, target cells in CIC structures can be found inside apparently non-phagocytic host cells that facilitate migration, division, and even escape from internalization into the host cells (7). In 2007, Overholtzer et al. reported that extracellular matrix detachment in cancer cells promote CIC formation via contractile forces associated with adhesive junction formation. This process involves the junctional proteins E-cadherin and β-catenin and is dependent on actomyosin contractility via Rho-associated coiled-coil-containing protein kinase (ROCK) activity in the target cells (1). This finding suggests target cell invasion as opposed to engulfment and has been confirmed in several studies (9, 10).

The above studies indicate that CIC structure formation is affected by modulation of the tumor microenvironment (TME), which may lead to changes in cellular characteristics. In this narrative review, we summarize the role of CIC in various cancer treatment strategies, that is, the changes and crosstalk of molecules and proteins surrounding CIC in the TME and the role of these factors in contributing to therapeutic resistance acquisition.

## 2 CIC and mutant p53

In many human cancer cells, p53 is mutated, leading to p53 expression loss or expression of a mutant p53 protein (11). Mutant p53 has been shown to not only lose wild-type function but also gain oncogenic traits, such as invasion and metastasis (12, 13). A previous study has indicated the role of mutant p53 in facilitating CIC structure formation and promoting genomic instability. In their xenograft mouse model, no growth advantage was found for mutant p53 cells compared to p53 null cells, but the mixed population of mutant p53 and p53 null cells had a growth advantage and the highest number of CIC structures, indicating that heterogeneity drives CIC formation (6). In addition to Tp53 mutations, Hayashi et al. reported that the genetic features of *KRAS* and *Myc* amplification were significantly associated with entosis in human pancreatic ductal adenocarcinoma (PDAC) tissues and were independently associated with a poor prognosis (14).

## 3 CIC and inactivated CDKN2A

Another tumor suppressor involved in entosis is CDKN2A, a cell cycle regulator and tumor suppressor gene in external cells. Liang et al. reported that CDKN2A inactivation promoted entosis and CDKN2A expression and was inversely correlated with CIC formation in breast cancer (BC). Specifically, they found that inhibiting CDKN2A effectively promoted homotypic CIC formation, whereas ectopic overexpression of p16INK4a or p14ARF, two proteins encoded by *CDKN2A*, significantly suppressed CIC formation in MCF7 cells. Regulation of CIC formation by CDKN2A is closely correlated with the subcellular E-cadherin redistribution, F-actin rearrangement, and reduced myosin light chain 2 (p-MLC2) phosphorylation, consistent with the fact that CDKN2A expression imparts cell winner (outer cell) identity in the cell competition assay (15). Moreover, expression of *KRAS^V12^* and loss of *CDKN2A* have been reported to downregulate myosin and thus cause outer cell deformability in CIC structures (16). Overexpression of *KRAS^V12^* and *c-Myc* has also been reported to promote the formation of CIC structures (8, 14, 17).

Therefore, entosis may cause the selection of “winner” tumor cells that have acquired mutations in *Myc*, *KRAS*, *CDKN2A*, and *p53*, leading to heterogeneity in the TME.

## 4 Molecular changes on surrounding CIC in the TME

The molecular changes required for invasion can also be found in entotic cells, including a rearranged cytoskeleton, ezrin expression, and increased ROCK activity. Cano et al. suggested that cannibalism, which they found to be ROCK- and β-catenin-independent, coincided with the inability of cells to undergo TGF-β-induced epithelial-to-mesenchymal transition (EMT) changes required for pancreatic cancer metastasis. These data suggest a pro-tumorigenic function for entosis and an anti-metastatic and anti-tumorigenic function for cannibalism (18). Moreover, the TME contains stromal cells, fibroblasts, and immune cells that induce entosis and can be modulated by tumor cells to produce large amounts of growth factors and cytokines (19).

Wang et al. reported the histochemical observation of heterotypic CIC between epithelial cells and lymphocytes in a wide range of colorectal tissues from colitis to colorectal cancer (CRC). Furthermore, the formation of CIC structures was increased in colorectal cancer tissues treated with high concentrations of the inflammatory mediator IL-6 compared to those treated with low concentrations of IL-6. These CIC structures are formed between tumor cells and cytotoxic T cells mostly through emperitosis. In addition, they found that IL-6 within the TME also promotes CIC formation by upregulating the expression of the cell adhesion molecule ICAM1 and increases inner CD8+ T cell motility via the activations of signal transducers and activators of transcription (STAT)3/5, extracellular signal-related kinase (ERK), and Rho-ROCK signaling pathways (20).

Furthermore, Ruan et al. reported that entosis was suppressed by knockdown of IL-8 and significantly enhanced by recombinant IL-8 treatment. This is related to the regulation of intercellular adhesion and the expression of adhesion molecules by upregulation of P-cadherin and γ-catenin. It is well-known that IL-8 regulates the inflammatory response. Then, neutrophils are recruited as inflammatory cytokines that regulate entosis (21).

**Figure 1:**
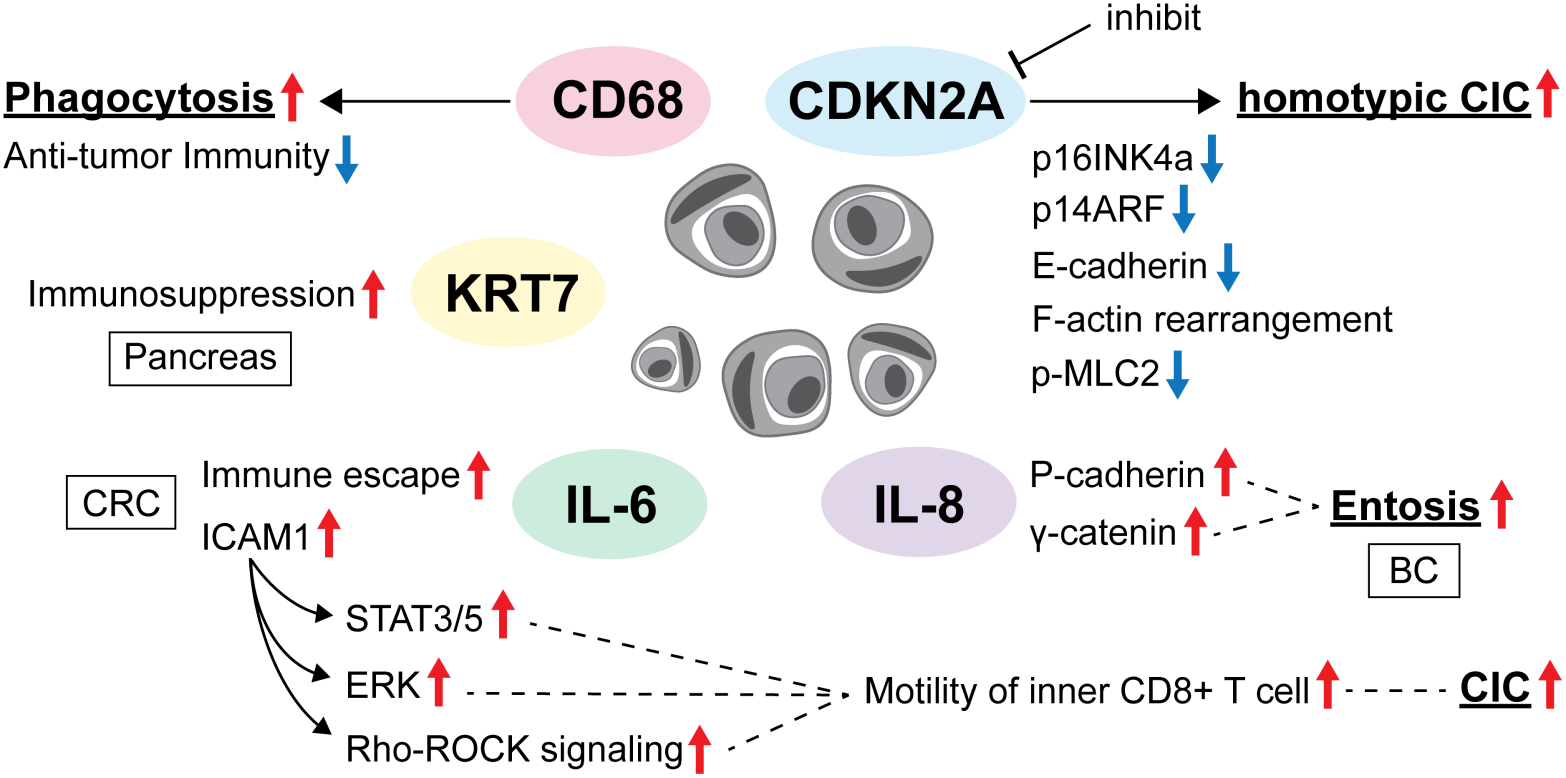
The evading strategy of CIC from anti-cancer therapies.

Overall, evidence on molecular changes has shown that the CIC structure has specific effects on cell metabolism and cell death, leading to tumor cell survival (**Figure 1**). These cellular and molecular changes may lead to therapeutic resistance.

## 5 Evading from anti-cancer therapies

During cell division, a group of proteins, including RhoA and myosin, cause cells to become rounder and stiffer. Durgan et al. suggested that this allows dividing cells to force their way into other cells, which is the first key stage of entosis. As cancer cells frequently divide, this form of cell cannibalism may lead to the destruction of cancer cells by their healthy neighboring cells. This reveals an unexpected link between cell division and cannibalism, which is relevant to both cancer progression and chemotherapy resistance (22). The internalized cells may be protected from harmful environmental factors, such as chemotherapy and other unfavorable conditions induced by anticancer drugs, by endocytic vacuoles formed within the host outer cell. The internalized cells then leave the outer cells intact. This results in anti-cancer drug therapy failure and cancer recurrence.

There are three major therapeutic strategies for cancer; however, a certain percentage of these strategies are resistant, and overcoming this is essential for improving the overall therapeutic effect of cancer treatments. Here, we summarize the role of CICs in chemotherapy, radiation therapy, and immunotherapy that result in cytotoxicity and discuss strategies to overcome resistance to them.

### 5.1 Evading from Anti-cancer drugs

Cancer cell cannibalism induces transient polyploidy, thereby facilitating cancer cell survival during anti-cancer therapy (23). In BC, paclitaxel and taxol promoted mitotic rounding and induced subsequent entosis in MCF7 cells (24). Resistance to chemotherapy has been achieved by heterotypic cannibalism; Bartosh et al. showed that heterotypic cannibalism (mesenchymal stem cell/mesenchymal cell incorporation) may induce BC cell dormancy and make them resistant to anti-cancer drug therapy (25). In prostate cancer (PC) cells, entosis is an escape mechanism from the anti-cancer effects of the tyrosine kinase inhibitor nintedanib. Entosis of PC cells by nintedanib occurs via inhibition of phosphoinositide 3-kinase (PI3K)/cell division cycle 42 (CDC42), followed by upregulation of E-cadherin and ROCK signaling pathways (26). Wen et al. identified that androgens also enhance entosis development and play a negative role during PC progression by influencing entosis by modulating the Rho/ROCK pathway (27). On the other hand, only one study of pancreatic cancer has associated CIC structure with the suppression of cancer metastasis, with evidence to the contrary for other cancer types (18). Although there is no additional evidence as to why only pancreatic cancer exhibits the above phenomenon, the fate of entosis may be influenced by tumor cell interactions and the TME, and the tumor extracellular matrix should always be considered.

There is still a paucity of *in vitro* and *in vivo* biological evidence as to whether CIC structures are truly chemotherapy- and targeted therapy-resistant, and it is desirable to build more evidence for anti-cancer treatment in the future. Therefore, it is necessary to establish useful and easy markers for CIC evaluation.

### 5.2 Evading from radiotherapy

Given the difficulty in establishing a CIC model, it remains challenging to evaluate the therapeutic effect of radiation or its resistance in tumor cells that form CICs in a strictly therapeutic manner. An *in vitro* study reported that irradiation triggered and promoted the formation of CICs. They indicated that decreased cell viability and TME modulations caused by irradiation could trigger non-professional phagocytosis (28). This result implies that tumor cells shift to escape irradiation to protect themselves and rather toward tumor progression. In contrast, using 601 human tissue specimens from 147 BC patients who participated in an institutional accelerated partial breast irradiation phase II trial, the group reported that CIC-positive patients had a good prognosis in terms of local recurrence-free and disease-free survival, but a poor prognosis in terms of metastasis-free survival. Furthermore, subgroup analysis indicated a correlation between a high proliferation index and high CIC rates, with CIC having the highest prognostic value in younger BC patients (29).

Schenker et al. investigated the contribution of CIC to the prognosis of head and neck squamous cell carcinoma (HNSCC) using pre- and post-radiochemotherapy (RCT) biopsy samples and revealed that CIC is a significant predictor of overall survival in pre-RCT biopsies but not in post-RCT biopsies. They speculated that CICs impairs the production of damage-associated molecular patterns (DAMPs) by capturing necrotic cells that inhibit an adequate anti-tumor immune response, which is reflected in the poor prognosis of patients with a high incidence of CIC (30).

In fact, the results of clinical studies sometimes do not agree with those predicted from *in vitro* data, indicating that using CIC alone as a direct predictor of prognosis for radiotherapy may not be promising. Further *in vivo* studies will reveal the actual biology of CIC formation in the TME, taking into consideration the TME heterogeneity.

### 5.3 Evading from immunotherapy

Several authors have discussed that various engulfment mechanisms resemble autophagy in cellular nutrition, in addition to protecting tumor cells from immune surveillance and influencing cancer development (31, 32). Gutwillig et al. investigated tumor cells that evade immunotherapy by generating unique transient CIC structures that are resistant to T cells and chemotherapy. They showed that while the outer cells are often killed by reactive T cells, the inner cells remain intact and disseminate into a single tumor cell when T cells are no longer present. Moreover, this effect is mediated primarily by IFNγ-activated T cells, which then induce the phosphorylation of the transcription factor STAT3 and early growth response-1 (EGR-1) in tumor cells. Their work showed the possibility of changing cold immune tumors to hot tumors (33).

Heterotypic CIC structures indicate the formation of CICs between immune cells and tumor cells, which serve as a mechanism of immune evasion that promotes cancer progression. Choe et al. identified an association between heterotypic CIC structures and anti-cancer drug resistance in CICs formed from NK and cancer cells. They reported three important findings: (i) cancer cells forming heterotypic CICs showed lower reactivity to NK cytotoxicity and higher proliferative capacity than non-CIC cancer cells; (ii) after anti-cancer drug treatment, cancer cells forming heterotypic CICs showed higher resistance to anticancer drugs than non-CIC cancer cells; and (iii) more CIC structures were observed in cancer cells treated with anti-cancer agents than in the non-treated group. These results suggest an underlying mechanism of immune evasion in heterotypic CICs and provide insight into anti-tumor drug resistance in cancer cells (34). In addition, Su et al. reported that CIC formation contributes significantly to the death of host tumor cells, but not to the death of internalized immune cells. This is a typical feature of NK cell-mediated killing and is superior to typical methods that exhibit an extracellular cell-killing approach. They identified CD44 on tumor cells as a negative regulator of intracellular immune killing via inhibition of CIC formation. Mechanistically, CD44 antagonizes NK cell internalization by reducing N-cadherin-mediated intercellular adhesion and enhancing Rho GTPase-regulated cell stiffness, and blockade of CD44 signaling results in the suppressive effects of NK cells on tumor growth associated with increased heterotypic CIC formation. This result implicates the therapeutic target potential of CIC as local immunotherapy (35). **Figure 1** shows the relationship between CIC and evasion of anti-cancer therapies.

## 6 Factors involved in the effectiveness of antitumor immunity

CD68 plays a critical role in promoting cancer cell phagocytosis, is upregulated in various types of cancer, and is a hallmark of poor antitumor immunity and adverse prognosis. Zhang et al. focused on CD68 expression in various types of cancer and reported that high CD68 levels in tumor samples correlated with adverse prognosis in glioblastoma, kidney renal clear cell carcinoma, lower-grade glioma, hepatocellular carcinoma, lung squamous cell carcinoma, thyroid carcinoma, and thymoma. Although the clinical prognosis and immune infiltration associated with high CD68 expression levels vary by tumor type, inhibition of CD68-dependent signaling may be a promising therapeutic strategy for cancer immunotherapy (36). Along with CD68, transmembrane protein TM9 is involved in phagocytosis (37). Song et al. constructed a pancreatic cancer prognostic model based on four CIC-related genes and showed that the high-risk group had a worse prognosis, higher tumor mutation burden, and lower immune cell infiltration than the low-risk group. KRT7, the most important risk gene in this model, was significantly associated with poor prognosis of pancreatic cancer in the TCGA dataset, and their cohort indicated that high KRT7 expression may be responsible for immunosuppression in the pancreatic TME (38).

**Figure 2:**
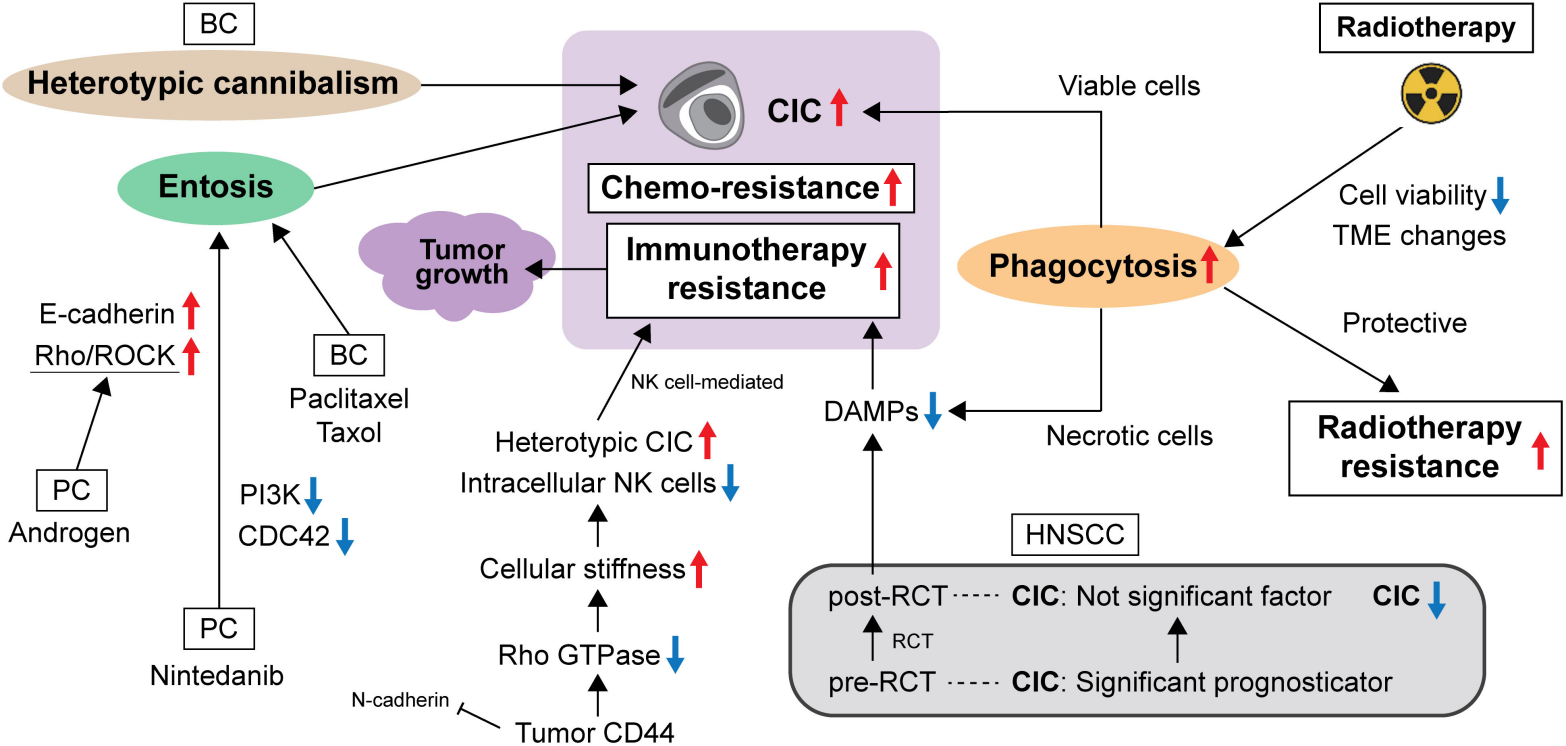
Molecular changes surrounding CIC in the TME.

Wang et al. suggested that heterotypic CICs formed by CRC cells and lymphocytes contribute to tumor escape from immune surveillance, which can be facilitated by IL-6 and may represent a previously undescribed pathway for tumor cells to evade host anti-tumor immunity. IL-6 in the TME also enhances tumor cell autophagy and promotes tumor cell survival in CIC while promoting the death of internalized lymphocytes (20). IL-6 inhibition may indirectly prevent CIC formation and eventually improve prognosis (**Figure 2**).

## 7 Discussion

Some researcher have found entosis as a type of regulated cell death debatable since the one keeps invading live cells inside the host cell. According to the Nomenclature Committee on Cell Death 2018, as a formal definition of the CIC structure at this time, they propose to define entotic cell death as a form of regulated cell death that originates from actomyosin-dependent cell-in-cell internalization and is executed by lysosomes (39).

Understanding the role of CIC in cancer cells is a major challenge in oncological biology and physiology. Larger cancer patient studies, a better understanding of the consequences of chemotherapy on CIC, biomarkers for each CIC formation process as well as the development of automated CIC detection in histological sections of tumor samples are required to use CIC presence as a clinical diagnostic tool in the future. In addition, in the TME, the interaction between CIC and surrounding tissues (e.g., cancer-associated fibroblasts, matrix metalloproteinases, tumor-infiltrating lymphocytes, etc.) has not been revealed so far, and this clarification can contribute to understanding the real role and function of CIC.

Again, Cano et al. indicated a protective role of homotypic CIC structures in PDAC and identified Nupr1 as a molecular regulator of this process (18). Interestingly, unlike cancers of other organs, CIC frequency and prognosis are inversely correlated in PDAC. A more detailed understanding of the biological mechanisms of Nupr1 involvement in CIC formation will emphasize the role of CIC and greatly contribute to the development of CIC-based therapies.

In addition, CIC-targeted cancer therapies using exosomes have been proposed. Exosomes and other extracellular vehicles can deliver agents with a defined anti-tumor activity (40, 41). At the same time, the cell ingestion of exosomes and the vehicles may be facilitated in cancers, representing one of the competent anti-tumor therapeutic approaches.

## Author contributions

KO obtained the research funding, wrote the original manuscript, revised the manuscript, and prepared the figures. SY provided ideas for research design. HF, TN, and SY have been previously studied in relation to the theme of this review article and provided findings. SY supervised the study. All authors contributed to the article and approved the submitted version.
